# The importance of post hoc approaches for overcoming non-response and attrition bias in population-sampled studies

**DOI:** 10.1007/s00127-015-1153-8

**Published:** 2015-11-28

**Authors:** Linsay Gray

**Affiliations:** MRC/CSO Social and Public Health Sciences Unit, University of Glasgow, 200 Renfield Street, Glasgow, G2 3QB UK

**Keywords:** Attrition, Non-response, Bias, Population based, Substance use

## Abstract

Population-based health studies are critical resources for monitoring population health and related factors such as substance use, but reliable inference can be compromised in various ways. Non-response and attrition are major methodological problems which reduce power and can hamper the generalizability of findings if individuals who participate and who remain in a study differ systematically from those who do not. In this issue of SPPE, McCabe et al. studied participants of the 2001–2002 National Epidemiologic Survey on Alcohol and Related Conditions, comparing attrition in Wave 2 across participants with different patterns of substance use at Wave 1. The implications of differential follow-up and further possibilities for addressing selective participation are discussed.

Valid estimation of the population prevalence of health-related behaviors including substance use are crucial for formulating and evaluating strategies aimed at improving and maintaining public health and wellbeing. However, accurate inference from population-based health studies is more often than not blighted by the problem of missing data [1]. Study samples are subject to “unit non-response” (or “non-participation”)—where people who have been selected for inclusion have not participated—and “item missingness” where participants have not provided data for all individual variables. In longitudinal studies, “attrition”—the loss of follow-up of cohort members over time—is yet another facet of the missing data problem. All three aspects lead to subsequent loss of power, increasing the chances of both type I (false positive) and type II (false negative) errors. The potential for bias is also elevated if certain sub-groups of individuals are systematically missing: under these conditions, associations among variables which may not be true can arise, and vice versa (internal validity is compromised) and the extent to which results are generalizable to the population (external validity) is threatened. The occurrence of missing data leads to bias in analysis unless the underlying mechanism of missing data is ‘missing completely at random’ (MCAR). MCAR relies on the probability of participation/of not answering a particular question/of dropping-out of follow-up being uncorrelated with individual characteristics. The assumption of MCAR in population-based studies is strict and usually implausible. Attrition tends to be cumulative over successive study waves and participant response levels have declined in recent decades [2], which is recognized as an escalating problem [3]. At the individual level, non-participation and attrition are typically associated with having lower socio-economic status and poorer health [4].

The paper by McCabe et al. [5] concerns the latter of the three missing data issues: attrition. Their study examined participants (all respondents at Wave 1) of the prospective 2001–2002 National Epidemiologic Survey on Alcohol and Related Conditions, comparing non-response in the 3-year follow-up Wave 2 interview among groups of participants categorized according to their use of an array of substances at Wave 1. The association between non-response at Wave 2 and substance use at Wave 1 was modeled in logistic regression models both univariably and also multivariably adjusting for socio-demographic characteristics. Socio-demographic characteristics themselves, along with their interactions with substance use were also examined. In addition, McCabe et al. explored the possibility of identifiable sub-groups of substance using participants by performed latent class analysis.

Relative to drug and alcohol users overall, non-users were found to have higher levels of attrition at Wave 2. However, once subset among users, those who use frequently have higher levels of attrition than those who use less frequently. Findings of higher levels of attrition among participants who were unmarried, older, male, Asian or Hispanic and with low education mainly concur with those in previous studies. As acknowledged, the utility of the latent class analysis to identify a potential substance use-based sub-group that had a particular propensity for attrition was diminished by the dominance of one class (comprising over 94 % of the participants) who typically drank occasionally but did not engage in the use of other substances. The substance use–attrition associations they identified parallel findings elsewhere of higher alcohol- and drug-related harm in survey non-participants relative to participants [6]. With differences of up to 200 % in the risk of substance-related harm, such coverage gap is a cause for concern, requiring robust means of resolution.

In general, attrition is not adequately addressed in longitudinal studies [7]. As McCabe et al. emphasize, their conclusions apply to attrition and not overall non-participation from Wave 1 and they rightly stress that factors related to attrition are not necessarily the same as those related to overall non-participation. The authors suggest a means to test the sensitivity of results to attrition in longitudinal data but stop short of providing a practical solution.

The methodology for addressing non-participation and attrition is advancing. Classic solutions encompass inverse probability weighting and multiple imputation—and sometimes combinations of the two techniques [8]—as well as the more recent harnessing of re-contact surveys [9] and of record-linked data [10], where available. In conjunction with sampling weights—as present in the McCabe et al. study—inverse probability weights are devised in surveys and longitudinal studies to adjust the relative contribution of each cohort member present in any particular sweep according to the similarity of their characteristics to those who dropped out. Such weights are usually defined as the inverse of the probability of response. For instance, if a study has lost more men than women, then the remaining men will be assigned larger weights than the women. Imputation is the substitution of some value for a missing data item and using an imputation model to repeat this over multiple data sets—multiple imputation—more fully allows for the uncertainty arising from single imputation. Multiple imputation is appealing as it makes estimation from the analysis model of point estimates and confidence intervals relatively straightforward.

The standard implementation of MI and IPW are appropriate if the data are missing at random (MAR) under which the probability of non-participation/item non-response/dropping-out are related to some of the observed characteristics of the respondent such as gender, social class and education. The third and last missing data mechanism is missing not at random (MNAR) in which the probability of non-participation/item non-response/dropping-out is related to unobserved characteristics. If data are MNAR then even implementing MI and/or IPW does not address the problem. In the case of MNAR, re-contact surveys or record-linked data can be harnessed to provide additional insights to missing data. Re-contact surveys involve non-participants being contacted anew and asked to respond to a small questionnaire consisting of a restricted number of key questions. Respondents to the re-contact surveys can be compared on the basis socio-demographic characteristics and, if appropriate, taken as representative of the non-participants for incorporation into the analysis. However, re-contact surveys are often bounded by the limited sets of questions and/or rely on reference to auxiliary information such as administrative data.

When attainable, record-linked administrative data offer a powerful means of bolstering population-based study data. Further to this, it can also be exploited with reference to general population data to inform [4] and potentially resolve [10] power-loss and distortion resulting from non-response, as follows. From comparisons of the composition of study respondents in terms of socio-demographic characteristics and substance-related harm with that of the population (from census and vital statistics), the numbers of missing study respondents within each socio-demographic/harm combination group can be identified. Observations for non-responders are then simulated within each stratum and their unknown substance use estimates can then by multiply imputed. This can be done whilst allowing substance use–harm associations to differ between respondents and non-respondents. This yields substance use estimates corrected for survey non-response.

McCabe et al. found evidence of interactions between the frequency of use of alcohol and socio-demographic factors. In light of this, they highlight the need for such interactions to be factored in when performing non-response adjustments. Indeed, when implementing multiple imputation, interactions, as well as any covariates present in the analysis model, should always be factored into the imputation model.

Along with a body of previous research, the McCabe et al. study points to the potential of tailored retention strategies according to substance use patterns in prospective studies. Whilst it is always better to secure the collection of the data than to use statistical methods to compensate for the fact that it is absent, approaches to post hoc addressing of unavoidable non-response and attrition are progressing and increasingly should be applied.
